# Zein-based nanoparticles: Preparation, characterization, and pharmaceutical application

**DOI:** 10.3389/fphar.2023.1120251

**Published:** 2023-02-01

**Authors:** Guijin Liu, Dongyu An, Junjian Li, Shiming Deng

**Affiliations:** Key Laboratory of Tropical Biological Resources of Ministry of Education, School of Pharmaceutical Sciences, Hainan University, Haikou, China

**Keywords:** zein, nanoparticle, drug delivery system, protein, preparation, characterization

## Abstract

Zein, as one of the natural and GRAS proteins in plant, is renewable, nontoxic, biocompatible and biodegradable. Over the past decade, many research efforts have been devoted to zein-based biomaterials for several industrial applications. Combining with research experiences in our research group, the preparation methods, characterizations and pharmaceutical applications of zein-based nanoparticles were summarized in this review. Zein NPs with different particle nanostructures have been prepared by chemical crosslinking, desolvating, dispersing and micromixing strategies. The pharmaceutical applications of zein NPs are mainly focus on the drug delivery. Zein NPs can improve the drug stability, increase the oral bioavailability, control the drug release and enhance the drug targeting, thereby improving the pharmaceutical effect effectively. More efforts are required to analyze the relationship among preparation methods, particle nanostructures and pharmaceutical properties in virtue of quality by design approach, and further promote the scale-up production and clinical application of zein NPs.

## Graphical Abstract

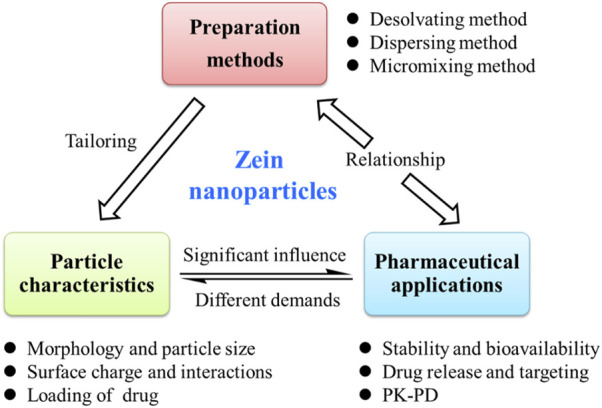

## 1 Introduction

Nanoparticles based on nanotechnology and biotechnology have attracted significant interest in drug delivery in the past decades (Mitchell et al., 2021). Various nanoparticles have been developed to improve the pharmacological and therapeutic properties of conventional (“free”) drugs, including poor aqueous solubility, instability, low bioavailability, serious adverse effects, and lack of targeted delivery. Among the available materials for preparing nanoparticles, protein provides numerous advantages over lipid, carbohydrate, synthetic polymer and inorganic colloidal carriers namely biodegradability, wide availability, environmental tolerance, and high drug binding capacity (Hong et al., 2020). A number of proteins including animal-derived proteins (e.g., albumin, gelatin, casein, lactoferrin, β-lactoglobulin, elastin, and lysozyme) and plant-derived proteins (zein, gliadin, glutenin, walnut protein isolate, and soy protein isolate) have been widely used as main natural backbones for the fabrication of nanoparticles. Compared to animal-derived proteins, plant-derived proteins are less expensive and reduce the risk of spreading diseases.

Zein, the major storage protein of corn, has been of scientific interest since its isolation in 1821 (Gorham, 1821). Zein is actually a heterogeneous mixture of different peptides of various molecular weights (MW), which can be classified into four classes: α-zein (two bands of average MW 22 and 24 kDa), β-zein (an average MW of 17 kDa), γ-zein (two bands of average MW 18 and 27 kDa) and δ-zein (an average MW of 10 kDa) (Shukla and Cheryan, 2001; Paliwal and Palakurthi, 2014). Structurally, zein is composed mainly of nonpolar and uncharged amino acids, such as glutamine (21%–26%), leucine (20%), proline (10%), and alanine (10%) (Pomes, 1971). The unbalance of amino acid composition leads to the poor water solubility and low nutritional value of zein. However, the peculiar amino acid composition also gives zein many favorable properties compared to other proteins. Zein behaves as amphiphilic but more specifically as a hydrophobic protein, which is insolubility in water except in the presence of alcohol (60%–95%), high concentration of urea, alkaline pH (≥11) or anionic surfactants (Evans and Manley, 1941; Manley and Evans, 1943). In contrast to drug delivery using hydrophilic proteins, zein has the capability of physically entrapping of hydrophobic compounds and yielding sustained drug release. Moreover, zein has a GRAS (Generally Regarded as Safe) status and readily self-assembles into nanoparticles with various structures depending on the solvents and the processing conditions (Wang and Padua, 2012b). Thus, zein has been extensively studied in terms of encapsulating and delivering bioactive ingredients.

Over the past decades, many research efforts have been devoted to the development and application of zein-based biomaterials. Some recent review articles have covered their applications in food and nutrition (Kasaai, 2018), phytochemicals delivery (Hassan et al., 2022), oral drug delivery (Irache and Gonzalez-Navarro, 2017), tissue engineering (Paliwal and Palakurthi, 2014) and other biomedical applications (Demir et al., 2017). In pharmaceutical field, zein has been investigated as excellent natural building blocks for preparing nanoparticles to delivery various active compounds. Combining with research experiences in our research group, the preparation, characterization and pharmaceutical application of zein-based nanoparticles (zain NPs) was summarized in this review.

## 2 Preparation of zein-based nanoparticles

**FIGURE 1:**
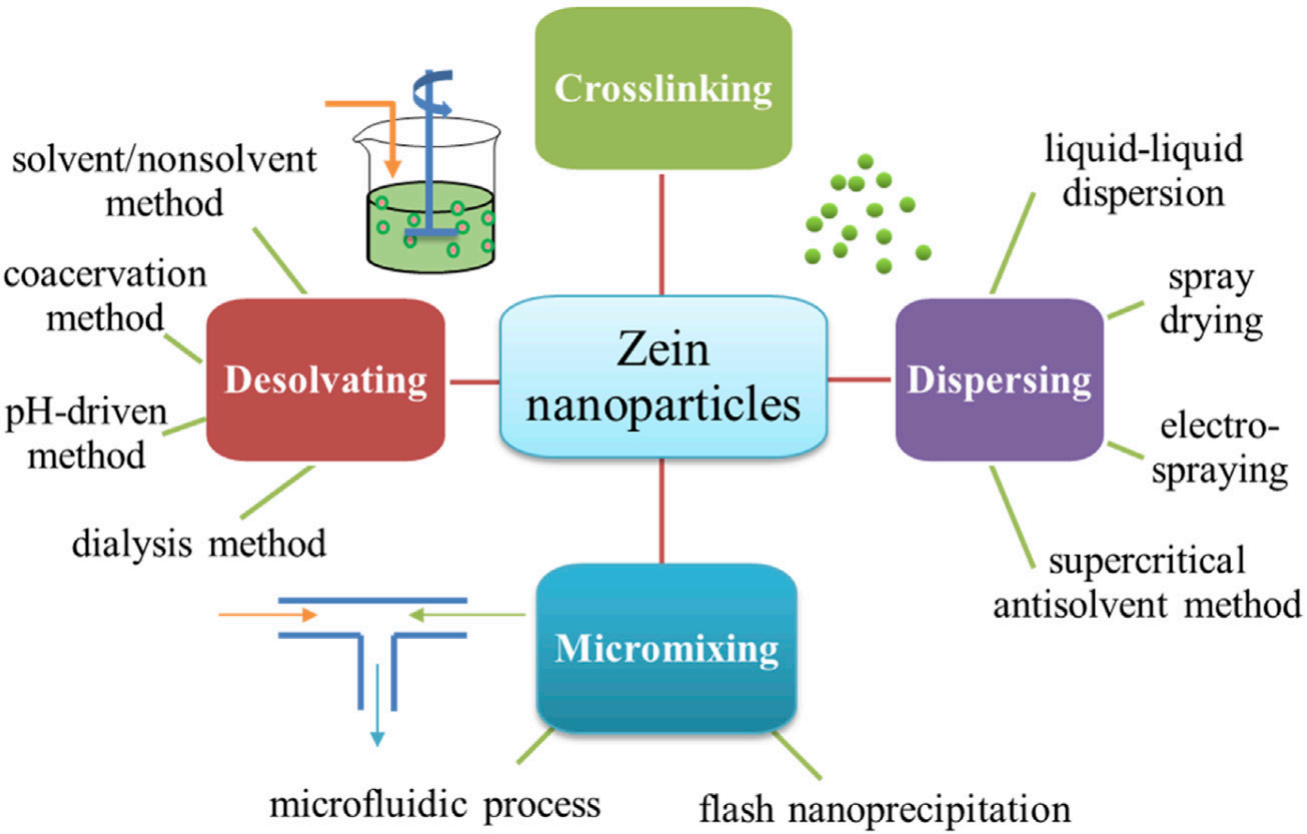
Preparation methods of zein-based nanoparticles.

The construction of zein particles can be traced back to the study of Suzuki et al., in 1989, that is, zein microspheres conjugated with antitumor drugs (mitomycinc, daunomycin hydrochloride, peplomycin sulfate, PS-K) have been prepared by glutaraldehyde mediated crosslinking reaction (Matsuda et al., 1989; Suzuki et al., 1989). There are abundant hydroxyl, amino and carboxyl groups on the side chains of zein molecule, which can be chemically coupled with hydrophobic or hydrophilic drugs by adding crosslinking agents, resulting in high encapsulation efficiency (*EE*). However, zein particles formed by chemical crosslinking are heavily aggregated and usually micro-scale. Also, crosslinking reaction may produce toxic or inactive drug derivatives, and the residues of toxic crosslinking agents and organic solvents may cause safety risks of final products. Therefore, it is imperative to develop alternative methods for preparation of zein NPs. Up to now various methods have been applied for preparing zein-based nanoparticles, and can be broadly classified as desolvating, dispersing and micromixing strategies (Figure 1). Each strategy has its own set of advantages and disadvantages, which have been summarized in Table 1.

**TABLE 1:** Advantages and disadvantages of the strategies for zein NPs generation.

Strategy	Principle	Method	Advantage	Disadvantage
Crosslinking	Crosslinking reaction between zein and actives	Glutaraldehyde mediated crosslinking reaction	High *EE*	Micro-scale size
			Encapsulation of hydrophobic and hydrophilic actives	Batch process
				Safety risks
Desolvating	Adding a reagent that can reduce zein solubility	Solvent/nonsolvent method	Low cost	Batch process
			Simple operation	Solvent consuming Tedious separation process
			Small particle size	
		Coacervation method	Simple operation	Micro-scale size
			Complex coacervation	Low *EE*
		pH-driven method	Avoid of organic solvents	Strong alkaline
			Composite zein NPs	Desalting process
		Dialysis method	High *EE*	Batch process
			Simple separation process	Time-consuming
Dispersing	Dispersing zein solution to small droplets	Liquid–liquid dispersion	Small particle size and narrow distribution	High-speed homogenizer
				Small-scale production
		Spray drying	Convenient operation	Micro-scale size
			Large-scale production	High temperature
		Supercritical antisolvent process	Semi-continuous process	Organic solvent
			Mild conditions	High pressure
				Specific equipment
		Electrospraying	Continuous process	High voltage
			High *EE*	Undesired structures at high zein concentration
			Simple equipment	
		Atomizing/antisolvent precipitation	Novel process	Interfacial resistance
			Core-shell structures	Batch process
			High *EE*	
Micromixing	Preparing in a confined volume	Flash nanoprecipitation	Controllable particle size Continuous process	Turbulent microfluidic
				Particle aggregation
		Microfluidic technique	Laminar flow regime	Pipeline blockage
			Uniform diameter	Small-scale production
			Continuous process	

### 2.1 Desolvating strategy

Desolvating is the main preparation strategy of zein NPs. It refers to adding a reagent that can reduce zein solubility in the solution, so that zein molecules can self-assemble into nanoparticles. Many variations of the desolvating technique exist, includes solvent/nonsolvent method, coacervation method, pH-driven method and dialysis method.

#### 2.1.1 Solvent/nonsolvent method

The most commonly used solvent/nonsolvent for the formation of zein NPs is the ethanol-water system. Under constant temperature, the solubility of zein varies between 2% and 60% (w/w) depending on the ethanol concentration (Mossé, 1961). The common procedure of solvent/nonsolvent method is as follows: 1) zein and drug are completely dissolved in 60%–90% alcohol; 2) the obtained solution is poured into deionized water (as nonsolvent), resulting in the supersaturation and self-assembly of zein molecules; 3) The solvent is removed by rotary evaporation, and zein NPs are collected after centrifugation, washing and drying. The solvent/nonsolvent process, also known as antisolvent precipitation or phase separation method, has been used to encapsulate ivermycin (Liu et al., 2005), ciprofloxacin (Fu et al., 2009), prednisolone (Lau et al., 2012), curcumin (Patel et al., 2010a), and many other drugs into zein NPs. In most cases, the obtained particles possess smooth surface, small and uniform particle size, and high drug loading capacity. For example, 5-fluorouracil/zein NPs were prepared using the phase separation method (Lai and Guo, 2011): under the optimal operating conditions, the surface of the nanoparticles was spherical and smooth, the particle size was 114.9 nm, and the *EE* and drug loading (*DL*) were 60.7% and 9.17%, respectively.

#### 2.1.2 Coacervation method

The preparation process of coacervation method is similar to that of solvent/non-solvent method. The difference is that zein and drug are first dispersed into water (or anhydrous ethanol), and then ethanol (or water) is added dropwise. According to the ternary phase diagram of zein solubility in ethanol and water, zein molecules can self-assemble to a coacervation state when ethanol concentration is lower than 40% or higher than 90% (Mossé, 1961). The simple coacervates of zein to encapsulate and release gitoxin in a controlled phase was prepared (Muthuselvi and Dhathathreyan, 2006). However, the obtained particles were micron-sized, and the *EE* was low (1.77%–20.98%). Crosslinking agents are often added to obtain crosslinked particles, which can moderately reduce the size of particles and increase the *EE* (Karthikeyan et al., 2012). Besides, simple coacervates of zein tend to aggregate during the storage due to their high surface hydrophobicity and neutral isoelectric point. Complex coacervation of zein with proteins [e.g., casein (Bao et al., 2021) and whey protein (Wei et al., 2021)], phospholipids [e.g., sophorolipid (Chen et al., 2020) and lecithin (Fu et al., 2021)], and polysaccharides [e.g., alginate (Khan et al., 2019) and chitosan (Pauluk et al., 2019)] is regarded as an effective approach to prevent hydrophobic aggregation and enhance stability.

#### 2.1.3 pH-driven method

pH-driven method is developed based on the solubility of zein in alkaline environment (pH ≥ 11.0) and insolubility in neutral or acidic aqueous solution. Briefly, zein is dispersed in deionized water, then the pH is adjusted to 11.0 or above with NaOH for zein dissolution, and then adjusted back to neutral or acidic by HCl. During the pH changing from alkalinity to neutrality, zein is protonated and gradually aggregated to nanoparticles (Yan et al., 2022). The pH-driven method has been widely used to prepare zein composite nanoparticles. For example, carvacrol-loaded zein/sodium caseinate composite nanoparticles were fabricated (Zheng et al., 2022): the obtained particles were spherical, the particle size was around 50–200 nm, and the *EE* was 77.96%–82.19%. Similarly, pH-driven zein/tea saponin composite nanoparticles were developed for encapsulation and oral delivery of curcumin (Yuan et al., 2021): the obtained particles were spherical, the particle size was around 100–250 nm, *EE* and *DL* were 83.73% and 22.33% respectively. Also, zein/propylene glycol alginate (Li et al., 2022), zein/pea protein (Zhao et al., 2022), zein/whey protein isolate (Zhan et al., 2020), zein/rhamnolipid (Dai et al., 2019), zein/whey protein isolate/carboxymethyl cellulose (Liu et al., 2022a) and other composite nanoparticles were prepared by pH-driven method. Coating and/or incorporating zein NPs with other hydrophilic or charged biopolymers can adjust their destabilization and aggregation behavior in aqueous suspension, thereby broadening their application in pharmaceutical and related industries. The progress in different methods and materials of surface-coated zein NPs and their encapsulated bioactive compound has been summarized recently (Yuan et al., 2022).

#### 2.1.4 Dialysis method

**FIGURE 2:**
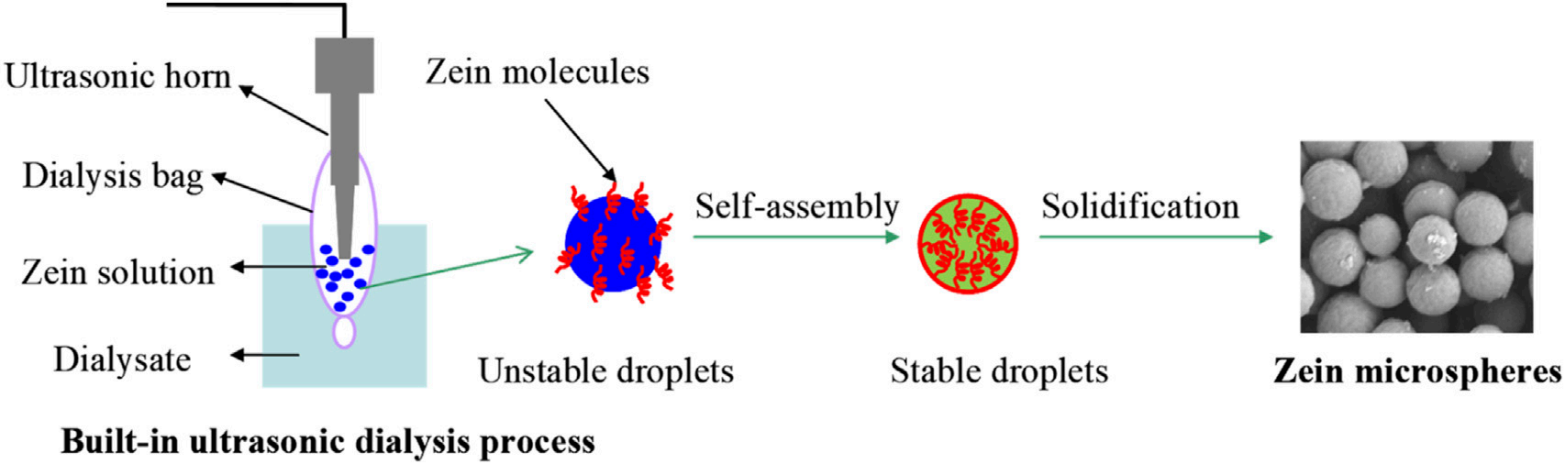
Self-assembly of zein microspheres using built-in ultrasonic dialysis process.

Dialysis method is a variant of solvent/nonsolvent method. Briefly, the dialysis bag containing an alcohol solution of zein and drug is placed in the dialysate (aqueous phase). When the solution in dialysis bag is contacted with the dialysate, diffusion of the solvent through the semipermeable membrane takes place, resulting in the change of the polar environment of zein, and the formation of zein NPs (Rodríguez-Félix et al., 2020). Solvent/nonsolvent method involves a large number of organic solvent, and requires a tedious separation process to remove the particles from the solvent. For dialysis method, solvent attrition and the addition of nonsolvent occur simultaneously, which avoids the additional operations to remove organic solvents after particle formation. In our previous work (Liu et al., 2016b), a novel built-in ultrasonic dialysis process (BUDP) was developed by combining the ultrasonic dispersing with dialysis technologies. Our results indicated that zein particles obtained by BUDP present much smaller particle size and narrower size distribution than those obtained from the conventional dialysis method. As shown in Figure 2, zein particles prepared by the BUDP possessed a spherical morphology with mean particle size from 400 nm to 2500 nm under different process parameters. Based on the BUDP, polydopamine coated zein-indomethacin particles with high *EE* of 89.1% were prepared for colon-targeted drug delivery (Wang et al., 2018).

### 2.2 Dispersing strategy

For conventional desolvating methods, zein NPs are formed from the bulk solution. The formed zein NPs are easy to aggregate to form the flocculates or precipitates during the drying process, which limit to some extent their usages in pharmaceutical industries. A general strategy to break this limitation is dispersing the stock solution to small droplets before the desolvating or drying process. The dispersing methods include but not limited to liquid-liquid dispersion, spray drying, electrospraying, supercritical antisolvent (SAS) process and atomizing/antisolvent precipitation (AAP) process. There are mainly two procedures regarding the particle formation: the breakup of stock solution into droplets, and the self-assembly and solidification of these droplets into nanoparticles during solvent attrition.

#### 2.2.1 Liquid–liquid dispersion

For the liquid–liquid dispersion, the stock solution of zein in 55%–90% aqueous ethanol is sheared to small droplets by a high-speed homogenizer before getting to the bulk deionized water. Due to the excellent miscibility of ethanol and water, ethanol in the dispersed droplets diffuses into the bulk water instantaneously, resulting in the solvent attrition. When the ethanol concentration in the dispersed droplets decreases below the solubilization limit of zein, these small droplets start to solidify and form nanoparticles. The size of zein NPs was typically 100–200 nm, and smaller particles were formed at a higher shear rate, and a higher ethanol concentration or a lower zein concentration in stock solutions (Zhong and Jin, 2009b).

#### 2.2.2 Spray drying

Spray drying is a frequently-used liquid atomization technology for producing a drying powder in pharmaceutical industry. It mainly involves four steps: feed atomization, air flow contact, drying and particle formation, and separation of product from the drying air (Mohammed et al., 2020). Atomization that involves dispersing the stock solution into small droplets is crucial to obtain a spray-dried product with desired properties. There are different types of atomizers, including rotary atomizers, pressure nozzles, pneumatic nozzles and sonic nozzles (Sosnik and Seremeta, 2015). Spray drying was applied as a commercially practical process to microencapsulate lysozyme in zein to achieve sustained release of the antimicrobial (Zhong and Jin, 2009a). The solution of zein, thymol, and lysozyme in 90% (v/v) aqueous ethanol was spray dried with a B-290 mini spray-dryer. The obtained particles were microcapsules with an encapsulation efficiency of 35.4%–49.1%. A similar method was adopted to obtain nisin/zein microcapsules with particle size less than 3 μm (Xiao et al., 2011). Spray drying has the advantages of convenient operation, short drying time, good fluidity and dispersion of products, and is suitable for large-scale production. However, it is not suitable for encapsulating temperature-sensitive actives, and the produced zein particles are usually microscale.

#### 2.2.3 Supercritical antisolvent process

SAS process, by exploiting the antisolvent effect of supercritical CO_2_ (scCO_2_), is a potential alternative to conventional antisolvent precipitation. The preparation process is as follows: 1) the drug and zein are dissolved in an liquid solvent that is miscible with scCO_2_; 2) the stock solution is sprayed through a micro-nozzle into the precipitation vessel containing scCO2; 3) when in contact with the scCO_2_, the solvent in the dispersed droplets diffuses into the scCO2 instantaneously, resulting in the solute supersaturation and formation of small particles. Lysozyme/zein particles were prepared *via* SAS process by using 90% ethanol in water as the liquid solvent (Zhong et al., 2009). However, the obtained particles were irregular, and the particle size larger than 50 μm. This may be due to the poor miscibility of 10% water and scCO2, resulting in a slow solvent attrition rate. In another study, coprecipitation of zein with diclofenac sodium at different polymer/drug ratio and concentration in the liquid solution were prepared by using DMSO as the liquid solvent (Franco et al., 2018). This time, microparticles with mean diameters ranging from 0.416 to 1.308 μm were obtained. Besides, lutein/zein NPs with mean particle size around 200–300 nm were successfully prepared *via* solution enhanced dispersion by supercritical fluids (SEDS, a modified SAS process) using acetone/DMSO (7:3, v/v) as the liquid solvent (Hu et al., 2012). SEDS process consists of a two (or three) coaxial passages nozzle to provide a simultaneous injection of the solution and scCO_2_. In this way, scCO_2_ acts both as an antisolvent and as a “spray enhancer” (Li and Zhao, 2017). ScCO_2_ as anti-solvent offers the advantages of mild operating temperature, being environmentally benign, less or solvent-free product. But the influence mechanism of operating parameters on the nanostructures and properties of obtained particles is complicated, involving the mutual interaction of fluid dynamics, phase equilibria and mass transport, and their influence on nucleation and growth mechanisms (Liu et al., 2013; Liu et al., 2015; Liu et al., 2021b). Thus, the preparation of zein NPs by SAS method needs further research.

#### 2.2.4 Electrospraying

Electrospraying (or electrohydrodynamic atomization) is an emerging and rather popular technique for obtaining the nanoparticles from biopolymers. In this process, the stock solution of zein is dispersed into tiny charged droplets under an electric field. Electrospraying is performed in a gas atmosphere, with the exception of the effective evaporation of solvent from the interface between the working fluids and their surrounding air environment (Malik et al., 2022). Being highly charged, the therapeutic molecules can easily be incorporated into zein NPs with high loading efficacy. On the basis of electrospraying, curcumin/zein NPs with a particle size of 175–900 nm and an *EE* of 85%–90% (Gomez-Estaca et al., 2012), and tamoxifen citrate/zein NPs with a particle size of 0.68–1.54 μm (Liu et al., 2018) were prepared. Besides, β-Carotene were encapsulated in zein matrix at micro- and nano-level through spray drying and electrospraying techniques, respectively, and the results proved that electrospraying had higher encapsulation efficacy than spray drying (Mahalakshmi et al., 2020). Electrospraying presents the advantages of simple equipment, high *EE* and small particle size, and does not require a tedious separation process to remove the particles from the solvent. However, the particle size is gradually enlarged with the increase of zein concentration (2.5%–15%), in which undesired collapsed and shrunken particles are yielded at high zein concentration (Gomez-Estaca et al., 2012).

#### 2.2.5 Atomizing/antisolvent precipitation process

**FIGURE 3:**
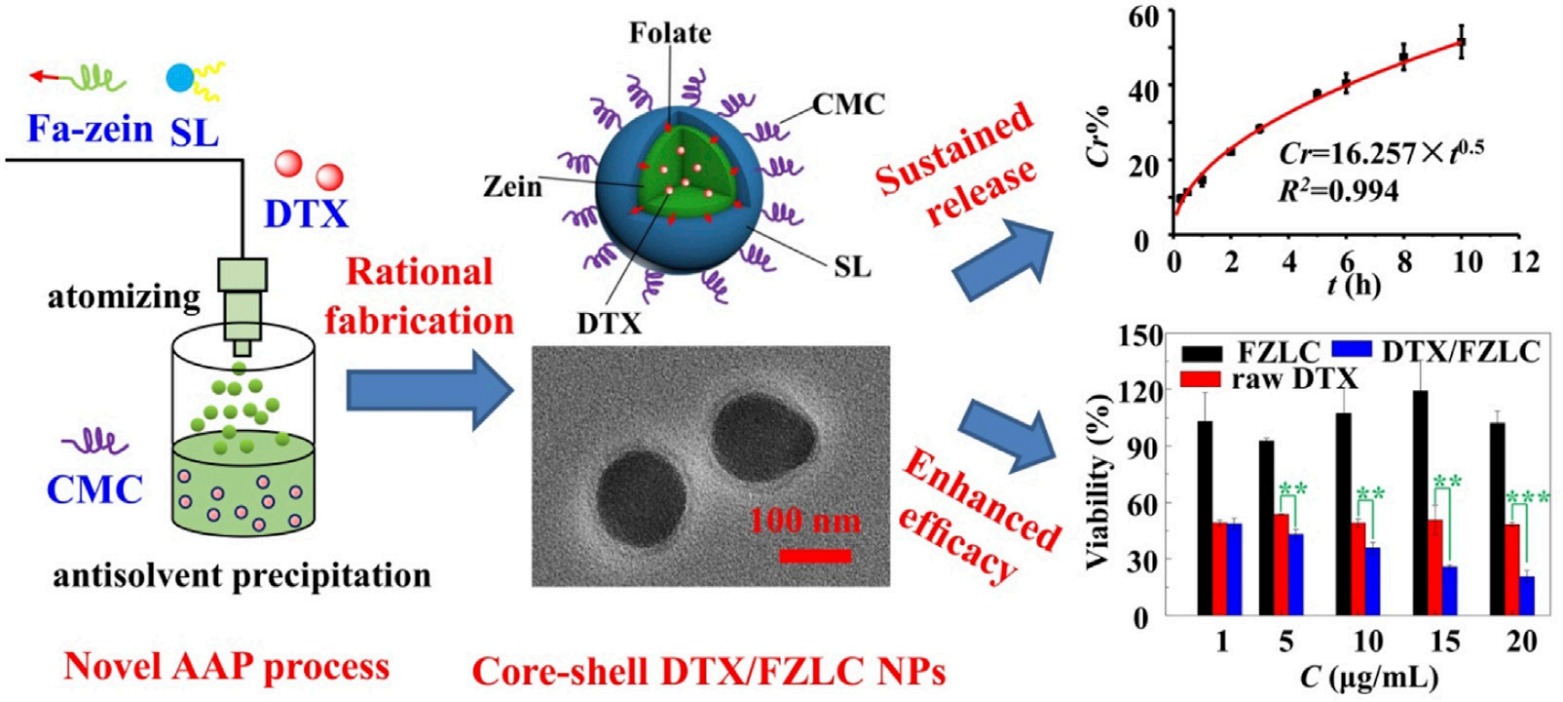
Rational fabrication and characteristic of docetaxel loaded folate-conjugated zein/soy lecithin/carboxymethyl chitosan core-shell nanoparticles (DTX/FZLC NPs) using a novel atomizing/antisolvent precipitation (AAP) process.

In our recent work, a novel and simple AAP process was established for the preparation of zein NPs by taking the advantages of atomizing and antisolvent self-assembly (Wu et al., 2022). On the basis of AAP process, folate-conjugated zein (Fa-zein)/soy lecithin (SL)/carboxymethyl chitosan (CMC) core-shell nanoparticles were successfully produced for the delivery of docetaxel (DTX), as shown in Figure 3. In brief, the solution of zein, SL and DTX in ethanol-water (70:30, v/v) was charged at a given flow rate, and atomized into fine droplets by compressed air through a nanosprayer, and then introduced into the antisolvent phase (CMC aqueous solution), and then self-assembled spontaneously into nanoparticles as the change of solution polarity from hydrophobic to hydrophilic. At a suitable condition, core-shell DTX/FZLC NPs with high *EE* (79.22% ± 0.37%), small particle size (206.9 ± 48.73 nm) and high zeta potential (−41.8 ± 3.97 mV) were obtained, which could increase the DTX dissolution, sustain the DTX release, and enhance the DTX cytotoxicity significantly. These results suggested that AAP process was suitable for the fabrication of complex zein NPs with multilayer core-shell structures. Further investigations and applications of AAP process are worth to be conducted.

### 2.3 Micromixing strategy

Different from the above methods, micromixing strategy prepares zein NPs in a confined volume, which allows a continuous operation and a better control on the fabrication of zein NPs. Thus, micromixing strategy is a continuous alternative to fabricate zein NPs. The representative methods are flash nanoprecipitation (FNP) and microfluidic technique.

#### 2.3.1 Flash nanoprecipitation

In the FNP process, the solution stream collides with a nonsolvent stream in a confined mixing chamber, which enables fast mixing on the order of millisecond and creates kinetically trapped NPs typically (Li et al., 2014; Saad and Prud’homme, 2016). Based on FNP process, resveratrol-loaded zein NPs with controllable particle size (50–108 nm) and narrow size distribution (Xing et al., 2022) were fabricated. Similarly, polysaccharide-stabilized zein NPs were fabricated for doxorubicin sustained release (Ye et al., 2022a), and PEG-b-PLA/zein NPs were fabricated for paclitaxel delivery (Ye et al., 2022b).

#### 2.3.2 Microfluidic technique

Microfluidic technique is also getting interest in the fabrication of zein NPs. Compared to FNP process, the flow in the microfluidic process is less turbulent due to the smaller channel diameter. The laminar flow regime of the solvents and predictable flow patterns produce consistent mixing conditions that result in particles of uniform diameter. A Y-junction microfluidics chip with staggered herringbone micromixers was successfully applied for the synthesis of zein NPs (van Ballegooie et al., 2019). And a T-junction configuration of the microfluidic chip was used to fabricate the zein-modified starch nanoparticle complexes for the encapsulation of nisin (Liu X. et al., 2022c). Both studies demonstrated that rapid and tunable microfluidic mixing could be used to continuously and reproducibly synthesize small and homogeneous zein NPs.

## 3 Characterization of zein-based nanoparticles

**FIGURE 4:**
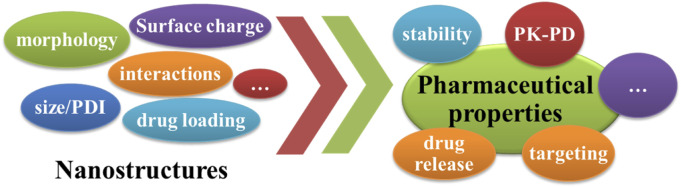
The nanostructures and pharmaceutical properties of zein-based nanoparticles.

For a drug delivery system, there are desired pharmaceutical properties, such as stability, drug release behavior, targeting, pharmacokinetic (PK) and pharmacodynamics (PD) characteristics. As shown in Figure 4, these pharmaceutical properties are directly influenced by the particle nanostructures, such as morphology, particle size, polydispersity index (PDI), surface charge and drug loading. Thus, it is necessary to characterize the particle nanostructures.

### 3.1 Particle morphology

Particle morphology can be observed using techniques such as scanning electron microscopy (SEM), atomic force microscopy (AFM) and transmission electron microscope (TEM). As an amphiphilic molecule, zein can self-assemble into spheres, micro-capsule, films, rods and particles with other morphologies (Wang et al., 2008; Wang and Padua, 2010; Wang and Padua, 2012a). In most cases, the obtained zein NPs are nanospheres with smooth surface. A possible mechanism for zein self-assembly from single molecules to nanospheres was proposed (Wang and Padua, 2012b). It mainly refers to four steps: α-helix to β-sheet transformation, packing of β-sheet into stripes, curling of stripes into rings, growing and rounding of rings into nanospheres. In some cases, hollow zein NPs are fabricated for their high loading capability, sustained release and low density. For example, hollow zein NPs were fabricated in a two-step process (Xu et al., 2011): preparation of sacrificial cores from sodium carbonate and precipitation of zein onto cores. The obtained hollow zein NPs had average diameters as small as 65 nm, and had 30% higher loading of metformin compared to the solid zein NPs. In another study, crocin loaded hollow dextran sulfate/chitosan-coated zein NPs were fabricated using sodium carbonate as a sacrificing template by a layer-by-layer self-assembly technique (Song et al., 2022).

### 3.2 Particle size

Particle size is the most important characteristic of nanoparticles in pharmaceutical industry. In general, nanoparticles are applicable to a wide range of biological targets due to their high intracellular absorption. But particles that are ‘too small’ exhibit poor encapsulation efficiency, may migrate from the site of injection, and may exhibit undesirably rapid release of their payload (Gaumet et al., 2008). Particle size is often determined using dynamic light scattering (DLS). Samples analyzed by DLS devices consist of particles that are well-dispersed in liquid media. DLS theory is based on the Brownian motion of particles in suspension, i.e., larger particles move slower than smaller particles at the same temperature. DLS can be applied over a wide size range, from nanometers to micrometers. In the case of polydisperse systems, one can simply determine the mean size and PDI from a cumulant analysis or, by a more complicated method (Glatter, 2018). However, when the particles are irregular and have a strong polydispersity, direct observation and measurement from their SEM or TEM images is suggested.

The particle size of obtained zein particles can vary from nanometers to micrometers under different conditions or preparation methods. The control of particle size is of prime importance in encapsulation and controlled delivery. The size increase of zein NPs during evaporation-induced self-assembly was measured by TEM, and modeled from the hydrophobic and hydrophilic contributions to the interfacial free energy from zein and solvent (Wang and Padua, 2012a). *R* ^2^ of 0.92 showed their model might allow the prediction of evaporation time and thus control over microsphere size. For self-assembly of zein NPs using BUDP, a semi-empirical model was developed for predicting particle size (*Dp*_*50*_) according to the analysis of influence mechanisms of operating parameters, i.e., $Dp_{50}=13904\cdot {\left(1+\frac{1220}{C}\right)}^{-1/3}\cdot \exp\left(0.049C\right)P^{-0.4}$ (*P* is ultrasound power, and *C* is zein concentration) (Liu et al., 2016b). This model could predict *Dp*_*50*_ at known experimental conditions or setting the process variables to achieve a given size, which was useful for a system capable of precise particle formation and scaling up BUDP. Besides, the modelling relationship between electrospraying product diameter and key experiment parameters were established by machine learning (Wang et al., 2022b), which could expedite the optimization process by accurately predicting particle diameter for both nano- and micron-sized particles.

### 3.3 Surface charge

Surface charge is a critical element in evaluating the dispersity and stability of nanoparticles *in vitro* and *in vivo*. It is often measured by the zeta potential measurement. In general, the absolute zeta potential values >30 mV can prevent the particles from clotting effectively, resulting in the good dispersity and stability of nanoparticles. At the isoelectric point, the zeta potential of particles is zero. Individual zein NPs are prone to destabilization and aggregation in aqueous and physiological fluids, because their neutral isoelectric point (pH 6.2) (Pascoli et al., 2018). The surface charge of zein NPs can be modified by adding surfactants, coating and/or incorporating with other biopolymers. For example, sodium caseinate was used to stabilize zein colloidal particles, which showed a shift of isoelectric point from 6.0 to around pH 5.0 (Patel et al., 2010b). In another study, the complex nanoparticles based on zein and fucoidan were developed to encapsulate resveratrol (Liu et al., 2022b). The obtained particles showed stable colloidal state and no visual aggregation at pH 2.0–8.0. These results are attributed to that fucoidan contains many sulfated groups and can be ionized at pH > 2.0, which provide electrostatic repulsion among particles.

### 3.4 Intermolecular interactions

The intermolecular interactions among different components of zein NPs are often characterized by circular dichroism spectroscopy, Fourier transform infrared (FT-IR) spectrophotometer and fluorescence spectrum. Circular dichroism spectroscopy is applied to characterize the conformational transitions of zein. When zein is combined with other components, the changes in the secondary structure of zein NPs can be observed (Liu et al., 2022b; Liu et al., 2022c). FTIR spectrum of zein shows peaks at around 3,310 cm^−1^ (O-H stretching), 1,660 cm^−1^ (amide I band) and 1,535 cm^−1^ (amide II band) (Sessa et al., 2013; Yin et al., 2015). Shift of these peaks provide evidence for the formation of hydrogen bonds, hydrophobic interactions, or crosslinking among different components of zein NPs. Fluorescence spectrum also can be applied to investigate intermolecular interactions. Zein contains a high proportion of tyrosine residues, which has a typical emission maximum around 304 nm after being excited at 280 nm. The interaction between zein and other compounds may lead to the micro-environmental alteration, which can be reflected through the fluorescence spectrum (Wei et al., 2019; Zhu et al., 2021).

In some cases, molecular dynamics (MD) simulation and molecular docking are used to analyze the intermolecular interactions. For example, the binding mechanism of zein with epigallocatechin-3-gallate (EGCG) was investigated *via* multi-spectroscopy and MD simulation (Liu et al., 2021a). MD simulation clarified that the van der Waals and electrostatic interactions were involved in binding of EGCG to zein, and the EGCG preferred to bind to the pocket of zein generated by residues Y171, Q174, L176 and L205. In another study, molecular docking was used to predict the precise binding sites and binding modes of ferulic acid to zein (Wang et al., 2022c), and the results revealed the binding stability under alkaline condition was stronger than that under acidic and neutral conditions.

### 3.5 Loading of drug

Zein molecule has a very special bricklike structure that provides sufficient space for drug entrapment (Geraghty et al., 1981; Zhang et al., 2016). The loading capacity of zein NPs is often evaluated by *DL* and *EE*, calculated using Eqs 1, 2, respectively:

$$DL=\frac{Amount\;of\;drug\;loaded\;in\;particles}{Amount\;of\;zein\;NPs}\times 100\%$$ (1)

$$EE=\frac{Amount\;of\;drug\;loaded\;in\;particles}{Amount\;of\;drug\;initial\;input}\times 100\%$$ (2)

The drug content was measured by a high-performance liquid chromatography system or UV-spectrophotometer. Due to the high hydrophobicity of zein molecule, zein NPs possess high loading capacity for hydrophobic drugs than hydrophilic and amphiphilic drugs (Karthikeyan et al., 2014).

**FIGURE 5:**
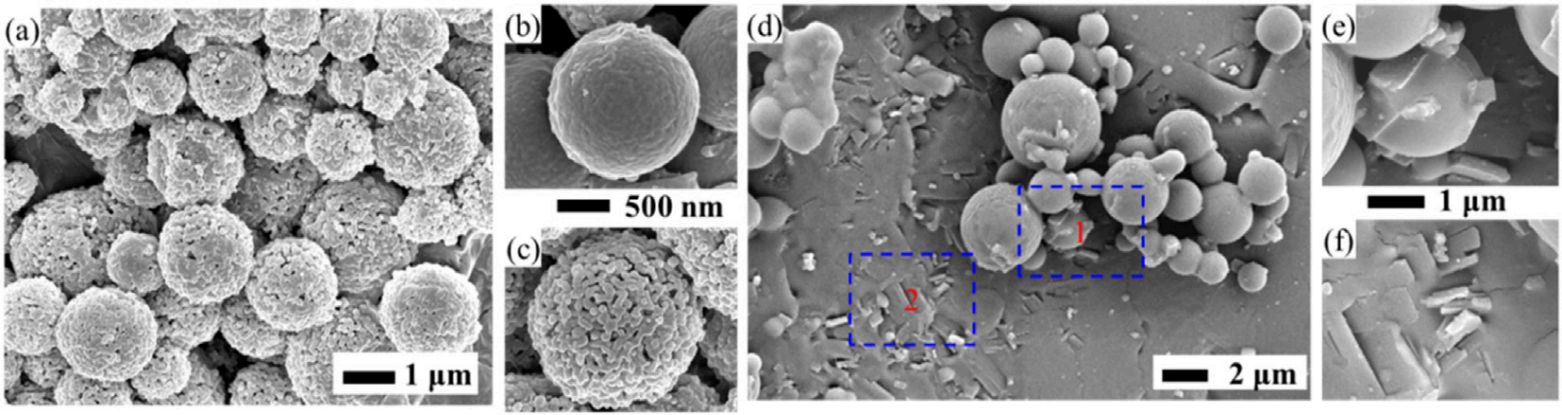
SEM images of 10-hydroxycamptothecin nanocrystals (HCPT NC)-zein particles after Na_2_CO_3_ treatment **(A)**, magnified images of HCPT NC-Zein particles before **(B)** and after **(C)** Na_2_CO_3_ treating; HCPT NC-Zein particles partially dissolved with ethanol–water solution **(D)**, magnified images of zone 1 **(E)** and 2 **(F)**.

The solid form of drug in zein NPs can be further analyzed by X-ray diffractometer (XRD) and differential scanning calorimeter (DSC). The XRD pattern of zein shows peaks at the diffraction angles of *2θ* = 9.2^o^ and 20^o^, where *2θ* = 9.2^o^ arises mainly from the α-helix structure of zein, whereas *2θ* = 20^o^ is thought to come from lateral α-helix packing (Chen and Subirade, 2009). Drug can exist in diverse solid forms, such as polymorphs, pseudo-polymorphs, co-crystals and amorphous solids (Liu et al., 2021b). In most cases, the drug is dispersed in zein NPs in an amorphous state. As an interesting study, drug nanocrystals (NC) were successfully incorporated into zein particles by combining the SAS process with BUDP in our previous work (Liu et al., 2016a; Wang et al., 2017), where the co-precipitation of 10-hydroxycamptothecin (HCPT) and zein prepared using the SAS process was dispersed into ethanol-water as the dialysis solution for BUDP. As shown in Figure 5, the obtained HCPT NC-zein particles were microspheres with a mean particle size around 1.0 µm, *DL* of 5.98% and *EE* of 95.68%.

## 4 Pharmaceutical application of zein-based nanoparticles

**FIGURE 6:**
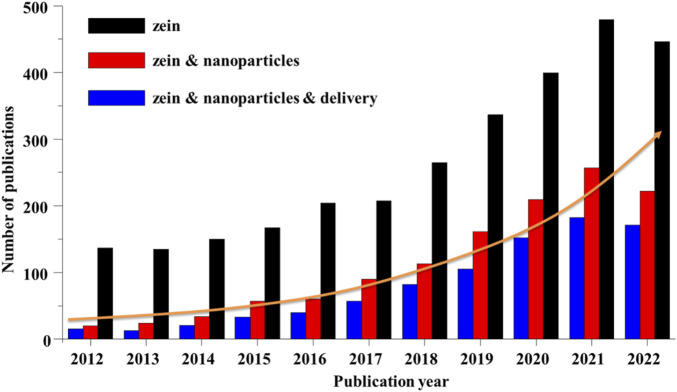
Number of publications on zein, zein & nanoparticles, zein & nanoparticles & delivery during the years 2012–2022. All the data were obtained through the Web of Science system as of 12 January 2023.

As shown in Figure 6, a search on the database Web of Science revealed that the number of published articles on zein has been constantly rising in the last decade. About half of the publications on zein are related to research for “nanoparticles” over the past 5 years. And the pharmaceutical application of zein NPs is mainly focus on the drug delivery. Zein NPs can not only protect the drug from the influence of the external environment, improve the drug stability and bioavailability, reduce the drug toxicity and side effects, but more importantly, have a good performance of controlling the drug release and enhancing the drug targeting, thereby improving the drug PK-PD effectively.

### 4.1 Improving the storage stability

Storage stability is vital to the shelf-lives of pharmaceutical products. Zein NPs usually have a smooth surface without pores, strong hydrophobicity and low permeability to oxygen, which can effectively prevent the contact of drug with external environment (e.g., light, oxygen, heat and humidity), thereby improving the storage stability. For example, the storage stability of 7,8-dihydroxyflavone (7,8-DHF) was strengthened greatly after encapsulation in zein NPs, especially with the addition of glycosylated lactoferrin (Chen et al., 2022). Similarly, zein NPs has been used to improving the storage stability of cannabidiol (Wang et al., 2022a), resveratrol (Khan et al., 2021), curcumin (Yuan et al., 2020) and others.

### 4.2 Increasing the oral bioavailability

The oral route is the preferred way for drug administration due to its advantages on patient compliance, safety, cost-effectiveness and feasibility. However, the complex nature of the gastrointestinal tract offers numerous obstacles, e.g., low pH of the gastric environment, presence of enzymes, intestinal flora and first-pass effect (Alqahtani et al., 2021). Due to their low oral bioavailability, many drugs have to formulate and administer as injections.

Zein NPs have already demonstrated an important potential to increase the oral bioavailability of both small and large molecules. Firstly, zein matrix is an excellent barrier for the protection of drugs (e.g., insulin (Reboredo et al., 2022), antigen (Hurtado-López and Murdan, 2006) and peptide (Khalid Danish et al., 2022)) in the gastrointestinal tract due to its water insoluble in a low pH environment (pH < 11.0). Secondly, the ability of zein in solid dispersion system can enhance drug dissolution rate of poorly water-soluble drug (Nguyen et al., 2017). In most cases, the drug is dispersed in zein NPs in an amorphous state. For the amorphous state, the drug molecules are not organized in a definite lattice pattern, and there is no crystal lattice energy to disrupt during dissolution, resulting in the highest level of dissolution (Liu et al., 2020). Thirdly, zein NPs possess a good bioadhesive nature with biological surfaces through hydrophobic interactions and hydrogen bonding. The mucoadhesive properties of zein NPs could lead to a slow drug release close to the cell membrane and a long retention time in the upper gastrointestinal tract for high drug absorption (Patel et al., 2010a). Moreover, their trans-mucus permeability and transmembrane transport can be further enhanced through the surface coating and modification (Bao et al., 2021).

### 4.3 Controlling the drug release

Drug release is a significant factor of nanoparticles for successful drug delivery. The drug release behavior is mainly dependent on drug solubility, diffusion, and biodegradation. It can be evaluated by a number of kinetic models, including statistical methods (e.g., exploratory data analysis method, repeated measures design, multivariate approach and multivariate analysis of variance), model dependent methods (e.g., zero order, first order, Higuchi, Korsmeyer-Peppas model, Hixson Crowell, Baker-Lonsdale model and Weibull model) and other model independent methods (Costa and Lobo, 2001; Dash et al., 2010).

Zein has been demonstrated to be an effective excipient in extensive studies for controlled drug delivery due to its hydrophobicity, biodegradability and biocompatibility. Zein is more suitable for sustained release of hydrophobic drugs than hydrophilic and amphiphilic drugs, due to the better binding affinity and greater interactions (hydrophobic, electrostatic, and hydrogen bonding) of hydrophobic drug with zein (Karthikeyan et al., 2014). Recently, the ability of zein in the controlled release of poorly water-soluble drugs has been reviewed (Tran et al., 2019). Moreover, for the controlled release of drug in specific environment, pH-responsive zein NPs have be prepared by coating with tannic acid (Liang et al., 2018), PDA (Zha et al., 2020), sodium caseinate and poly ethylene imine (Pourhossein et al., 2020). Also, there are few studies focus on the controlled release of zein NPs by other specific stimuli (Marín et al., 2018; Zhong et al., 2022).

### 4.4 Enhancing the drug targeting

Targeted drug delivery that refers to predominant drug accumulation within a target zone, is an effective approach to decreasing drug toxicity and enhancing therapeutic effects. The targeting of drugs can be viewed on two levels: organ targeting and cellular targeting (Koo et al., 2005; Manzari et al., 2021). The organ targeting is mainly dependent on the particle size, morphology and material properties of the carrier employed. Zein NPs have capabilities of size-dependent organ targeting through leaky tumor capillary fenestrations into a tumor vasculature according to the enhanced permeability and retention (EPR) effect (Maeda, 2015). For example, the organ targeting of 5-fluorouracil loaded zein NPs was studied (Lai and Guo, 2011): the targeting efficiency of drug in liver increased from 22.67% to 54.00% after the encapsulation, and the relative uptake rate (zein NPs/plain drug solution) was in the order of liver (2.79) > spleen (1.94) > plasma (1.09) > lung (0.70) > heart (0.34) > kidney (0.24), indicating that zein NPs was specific to the liver tissue. The development of magnetic NPs can further enhance the delivery and controlled release of a drug to the targeting organ, especially the fabrication of temperature and pH-responsive magnetic NPs (Marín et al., 2018; Pang et al., 2018).

**FIGURE 7:**
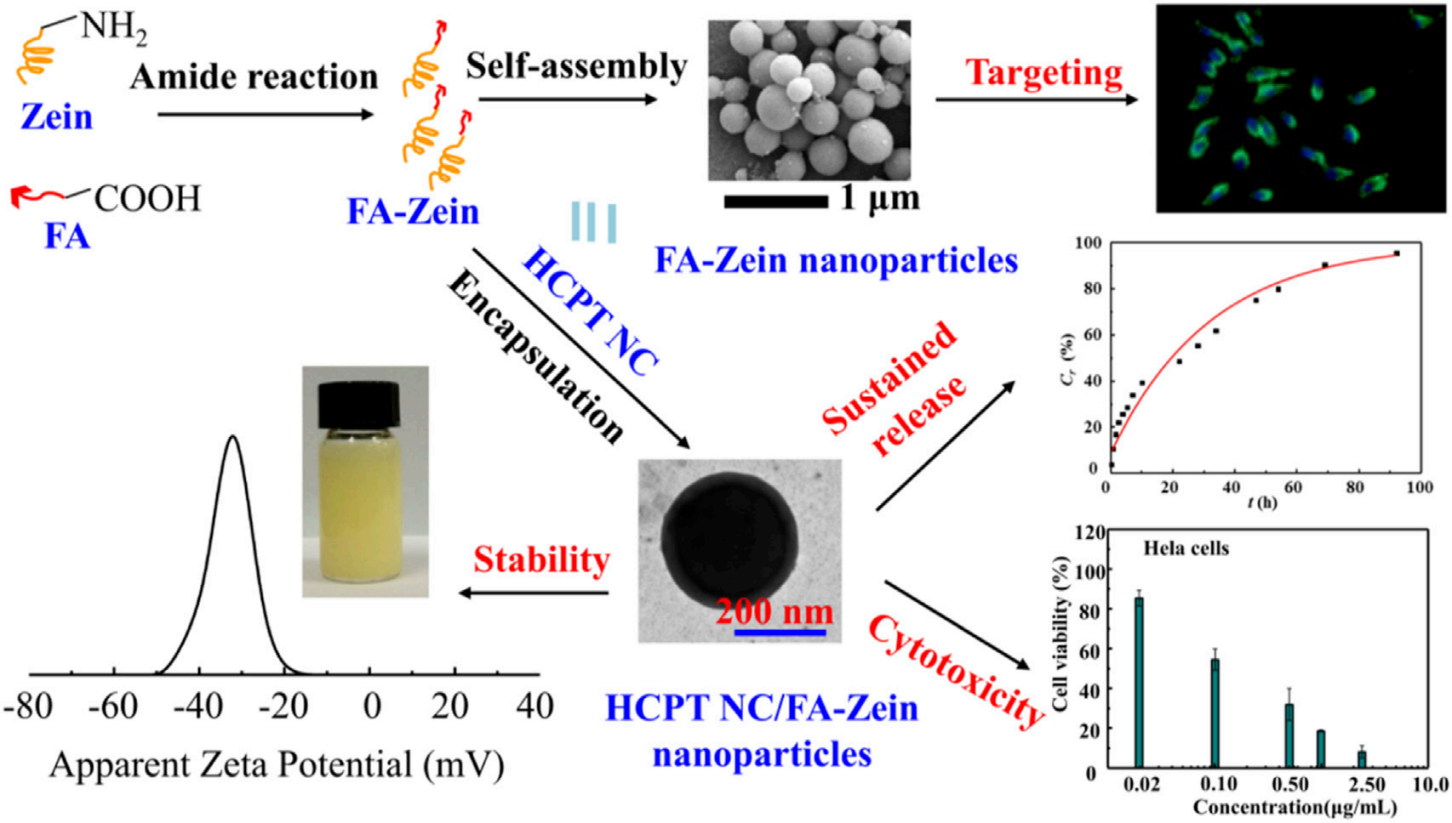
Synthesis and characterization of folic acid (FA) cross-linked zein nanoparticles for the targeted delivery of 10-hydroxycamptothecin (HCPT).

In spite of the preferred accumulation of zein NPs through organ targeting mechanisms, there is some degree of non-specific action in this method. It is of critical importance to develop the cellular targeting zein NPs through a more specific interaction at a molecular level between the carrier and the targeted cell (Byrne et al., 2008). There are abundant hydroxyl, amino and carboxyl groups on the side chains of zein molecular, which offer the possibility of conjugation and modification of zein molecules with targeting ligands or molecules (Allen, 2002; Xu et al., 2013). For example, hyaluronic acid (HA) cross-linked zein NPs were prepared (Seok et al., 2018; Zhang et al., 2020). Given the high targetability of HA to CD44 expressing cancer cells, the obtained HA-zein NPs could enter tumor cells effectively *via* a CD44 mediated endocytosis thereby enhancing the PK/PD. In our previous work (Liu et al., 2017), zein was decorated with folic acid (FA) for targeted delivery of HCPT. As shown in Figure 7, FA conjugation could not only facilitate the formation of small nanoparticles with good dispersity and stability, sustain the drug release, but more importantly improve the cellular up-take in folate receptor positive cells thereby enhancing the antitumor activity.

## 5 Future perspectives

Although zein NPs have made a great progress in their preparation, characterization and pharmaceutical application, they have yet to gain a foothold in the market up to now. Some issues remain to be considered before their scale-up production and clinical applications: 1) the effective strategy for the chemical modification and surface coating of zein NPs; 2) the precise preparation of zein NPs with desired nanostructures; 3) correlation of *in vitro* and *in vivo* characteristics; 4) evaluation of the PK-PD relationship and clinical feasibility.

**FIGURE 8:**
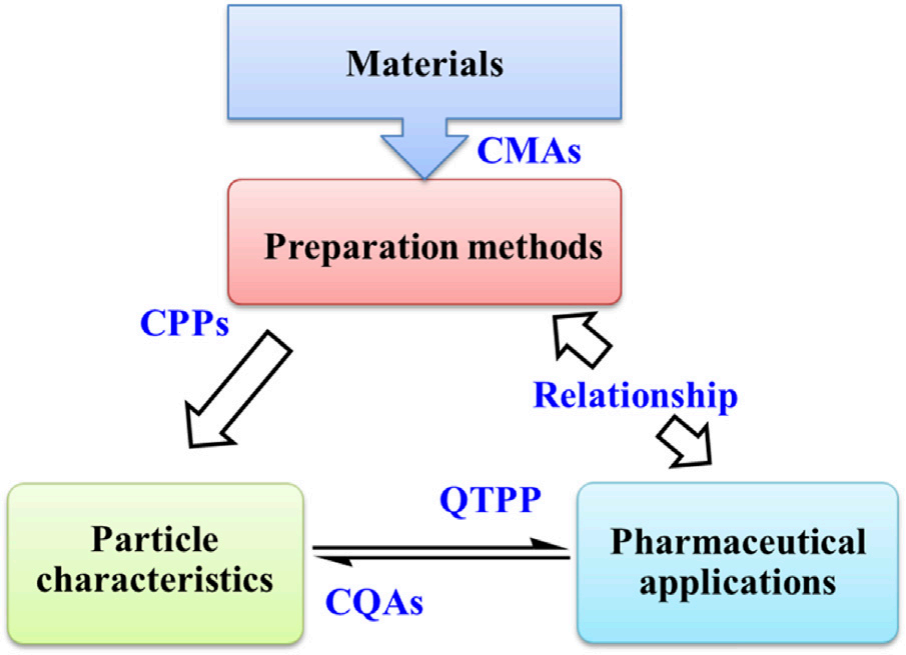
The relationship among preparation methods, particle characteristics and pharmaceutical applications of zein-based nanoparticles in virtue of quality by design approach. CMAs: critical material attributes, CPPs: critical process parameters, CQAs: critical quality attributes, QTPP: quality target product profile.

In Figure 8, a simple roadmap for the research and development of zein NPs is put forward on the basis of quality-by-design (QbD) approach. QbD approach is useful to the development of standardized procedures for getting the optimized product (Bastogne, 2017; Cunha et al., 2020; Soni et al., 2020). The quality target product profile (QTPP) of zein NPs can be specified firstly according to their pharmaceutical application. Then, critical quality attributes (CQAs) are selected from parameters that interfere with QTPP. In general, pharmaceutical properties are directly influenced by the particle nanostructures. CQAs of zein NPs can be further related to their nanostructures. After that, zein NPs with desired CQAs can be obtained by controlling the critical process parameters (CPPs) of preparation method and the critical material attributes (CMAs) of materials. And the design space of CPPs and CMAs is defined and optimized by risk assessment, design of experiment and process analytical technology.

## 6 Conclusion

Zein is a hydrophobic plant protein extracted from corn gluten meal. It possesses many favorable features as a natural building block for the development of drug delivery systems, e.g., a GRAS state, unique solubility and self-assembly properties. Zein NPs can be prepared by chemical crosslinking strategy, desolvating strategy (solvent/nonsolvent method, coacervation method, pH-driven method and dialysis method), dispersing strategy (liquid-liquid dispersion, spray drying, electrospraying, SAS process and AAP process) and micromixing strategy (FNP process and microfluidic technique). The obtained zein NPs exhibit different particle nanostructures, such as morphology, particle size, surface charge, intermolecular interactions and drug loading, which can well characterized by modern analysis methods. Zein NPs can be applied in pharmaceutical preparations to improve the drug stability, increase the oral bioavailability, control the drug release and enhance the drug targeting, thereby improving the drug PK-PD effectively. More studies in the future should deeply analyze the relationship among preparation methods, particle nanostructures and pharmaceutical properties in virtue of QbD approach.
